# Combination checkpoint blockade and laser interstitial thermal therapy in radiographically progressive non-small cell lung cancer brain metastases

**DOI:** 10.1093/noajnl/vdae207

**Published:** 2024-12-18

**Authors:** Aden P Haskell-Mendoza, Ethan S Srinivasan, Ariel T Gonzalez, Ellery H Reason, Joshua D Jackson, Ann Marie Flusche, Lucas P Wachsmuth, Emily Lerner, Delaney Underwood, Evan D Buckley, Saif E Zaidi, James E Herndon, Peter E Fecci

**Affiliations:** Duke University School of Medicine, Durham, North Carolina, USA; Department of Neurosurgery, Johns Hopkins University Hospital, Baltimore, Maryland, USA; Duke University School of Medicine, Durham, North Carolina, USA; Duke University School of Medicine, Durham, North Carolina, USA; Department of Neurosurgery, Duke University Medical Center, Durham, North Carolina, USA; Duke University School of Medicine, Durham, North Carolina, USA; Preston Robert Tisch Brain Tumor Center, Department of Neurosurgery, Duke University Medical Center, Durham, North Carolina, USA; Preston Robert Tisch Brain Tumor Center, Department of Neurosurgery, Duke University Medical Center, Durham, North Carolina, USA; Department of Biostatistics and Bioinformatics, Duke University School of Medicine, Durham, North Carolina, USA; Biostatistics Shared Resource, Duke Cancer Institute, Durham, North Carolina, USA; Preston Robert Tisch Brain Tumor Center, Department of Neurosurgery, Duke University Medical Center, Durham, North Carolina, USA; Department of Biostatistics and Bioinformatics, Duke University School of Medicine, Durham, North Carolina, USA; Preston Robert Tisch Brain Tumor Center, Department of Neurosurgery, Duke University Medical Center, Durham, North Carolina, USA

**Keywords:** brain metastases, immune checkpoint blockade, laser interstitial thermal therapy, non-small cell lung cancer

## Abstract

**Background:**

Laser interstitial thermal therapy (LITT) is a minimally invasive surgical treatment being employed frequently for radiographically progressive brain metastases. Considerable interest exists in combining LITT-mediated in situ vaccination to license immune checkpoint blockade (ICB). No studies have examined the clinical feasibility of this combination in brain metastases.

**Methods:**

All patients receiving LITT for radiographically progressive non-small cell lung carcinoma (NSCLC) brain metastases at a single center from 2015 to 2023 were retrospectively reviewed. Combination therapy was defined as ICB within 6 weeks of LITT. Clinical data, post-LITT freedom from local progression, and overall survival (OS) were collected. Adverse events (AEs) were evaluated according to Common Terminology Criteria.

**Results:**

Eighteen patients received LITT + ICB for a total of 19 lesions. The median time between therapies was 2.29 weeks (range 0.85–5.98). In comparison to NSCLC patients receiving LITT alone or with targeted therapy (LITT only) (*n* = 25), there was no decrement in procedural outcomes. Patients receiving LITT + ICB discontinued steroids at a median of 11 (4–147) days post-LITT vs. 24 (3–242) days for patients receiving LITT only (*P* = .62). At study cutoff, the local control rate was 18/19 (94.7%) lesions in the LITT + ICB group and 22/25 (88.0%) in the LITT only group. There were 3 and 5 AEs ≥Grade 3 in the LITT + ICB and LITT-only groups, respectively.

**Conclusions:**

Combination of LITT and ICB does not compromise procedural outcomes or time to steroid discontinuation in NSCLC. Prospective studies are needed to assess biomarkers of immune response.

**Key Points**

- No prior studies have examined a combination of ICB + LITT in brain metastases.
- Safety and procedural outcomes of combination LITT + ICB are similar to LITT without ICB.
- LITT for radiographically progressive metastases mediates excellent local control.

**Importance of the Study**

While there is considerable interest in using LITT to license immune checkpoint inhibition, no published study has examined this therapeutic strategy in the context of brain metastases, for which checkpoint blockade has FDA-approved indications. In a retrospective cohort of non-small cell lung carcinoma (NSCLC) patients receiving LITT + ICB, we showed that postoperative outcomes, including length of stay, discharge to home, intraoperative complications, and 30-day readmissions were similar to a comparable cohort of patients receiving LITT alone. Furthermore, patients exhibited high rates of steroid discontinuation and post-LITT local control. We also examined immune-related and central nervous system adverse events in this NSCLC cohort, with no concerning safety signals.

Laser interstitial thermal therapy (LITT) is a novel, minimally invasive neurosurgical procedure that has gained increasing use in the management of progressive or less easily accessed primary and metastatic brain tumors.^1,2^ During LITT, a laser fiber is advanced into a target lesion and used to deliver infrared light that is locally converted into thermal energy, producing coagulative necrosis; a stereotactic biopsy is frequently performed prior to LITT for tissue diagnosis.^3^ Extent of ablation is monitored via real-time magnetic resonance thermometry, allowing for controlled tissue damage intended to spare surrounding eloquent structures and white matter tracts.^3,4^ LITT has been shown to induce transient opening of the blood-brain barrier (BBB) in human and animal models in a manner sufficient to allow the delivery of molecules as large as immunoglobulins.^5,6^ Furthermore, LITT may serve as a form of in situ tumor vaccination, allowing for increased presentation of tumor neoantigens and release of damage-associated molecular patterns that elicit priming and activation of a T-cell mediated immune response.^3,7,8^ To date, the extent to which LITT may overcome the immunosuppressive mechanisms inherent to intracranial tumors remains incompletely characterized.^3,7^

Central nervous system (CNS) tumors are known to leverage several mechanisms for immune evasion and escape, including T-cell sequestration and exhaustion.^9,10^ Despite successes in other solid tumor types, immune checkpoint blockade (ICB) has failed in multiple randomized phase III trials for glioblastoma, although interest persists for neoadjuvant ICB administration prior to open resection.^11–14^ As clinical interest in LITT has increased, several early-phase trials investigating the combination of LITT and ICB in primary brain tumors have been initiated (NCT03277638).^15,16^ However, treatment of recurrent brain metastases and/or radiation necrosis (RN) following upfront stereotactic radiosurgery (SRS) +/− resection has recently become a key indication for LITT, with concomitant pre-LITT biopsy allowing for confirmation of viable tumor versus RN and subsequent adjustment of systemic therapies.^1,17^

Patients with brain metastases have frequently been excluded from pivotal trials in oncology, often in an attempt to obviate worsened survival in the setting of intracranial disease.^18^ Immune checkpoint inhibitors are among the few agents that have demonstrated efficacy in subgroups of patients with stable or treated CNS metastases, particularly non-small cell lung cancer (NSCLC) and melanoma.^18–20^ More recent studies have demonstrated favorable safety profiles of a combination of SRS and ICB, with some indication of combinatorial efficacy in specific disease histologies.^21,22^ Despite the increasing use of LITT for the management of radiographically progressive metastases, and further despite the standard employment of ICB for brain metastases, no studies have yet examined the use of a combination of LITT and ICB in this setting. To better establish the utility and safety of this treatment combination, we evaluated all patients receiving combination LITT and ICB at our institution, a high-volume brain metastasis and LITT center. To control for the impact of differing histologies, we compared NSCLC patients who received combinatorial LITT + ICB treatment versus LITT alone or with targeted therapy (LITT only), demonstrating parity in procedural outcomes, post-LITT steroid discontinuation, and freedom from local progression (FFLP), with comparable adverse event rates across groups.

## Materials and Methods

### Clinical Data

All patients receiving LITT for radiographically progressive brain metastases at a single institution from July 2015 to August 2023 were retrospectively reviewed in accordance with an IRB-approved protocol; consent was not required for participation. All patients who received ICB within 6 weeks before or after LITT, the maximum FDA-approved dosing interval, were included in the study.^23^ A control arm consisting of patients with NSCLC undergoing LITT who had never received ICB throughout their treatment course was constructed, though concomitant oral targeted therapy was allowed. All procedures were performed by the senior author. Baseline molecular and demographic data, systemic disease status, type of immunotherapy or targeted therapy received, and prior lines of local therapy were collected. Lesion measurements were obtained from radiographic notes or by the average of perpendicular diameters if not reported. The surgeon estimated % lesion ablation as approximated by the blue thermal damage threshold line collected from operative notes. Post-LITT outcomes were collected, with any immune-related adverse events (AEs) or CNS AEs reasonably suspected to be related to LITT during the study period graded according to Version 5.0 of the Common Terminology Criteria for AEs. Steroid discontinuation was assessed from the time of administration of procedural steroids (4–8-day taper as informed by symptoms) to the beginning date at which a patient remained off steroids for intracranial edema management for ≥4 weeks. This endpoint is similar to that of an ongoing prospective trial (NCT05124912). Overall survival (OS) was measured from the date of the index LITT case onward with patients censored at the last follow-up. For determination of FFLP, any LITT-treated lesion that underwent subsequent radiographic growth prompting a change in clinical management was considered progressive; for both FFLP and steroid discontinuation, patients were otherwise censored at death or last follow-up. No patient was excluded due to follow-up duration.

### Statistical Analyses

Within each group, continuous data were summarized as medians and ranges; groups were compared using Mann–Whitney tests. Categorical data were summarized as frequency counts and percentages; either a Chi-squared test or Fisher’s exact test (for expected *n* < 5) was used to compare groups. Time to steroid discontinuation, FFLP, and survival time were plotted using the Kaplan–Meier method and compared using log-rank tests. All statistical comparisons were two-sided. A *P*-value <.05 was considered significant. Analyses were performed in GraphPad Prism 10.0 (GraphPad Software) and R version 4.3.3 (R Foundation for Statistical Computing).

## Results

### Patient Cohort

A total of 27 patients received a combination of LITT and ICB to 28 lesions. Predominantly, these were patients with brain metastases: 19 lung primaries (18 NSCLC and 1 small-cell lung cancer [SCLC]), 3 melanoma, 2 renal cell carcinoma, and 1 pancreatic neuro-ectodermal tumor. Two patients with IDH-wild-type glioblastoma also received LITT + ICB. To compare outcomes in a more homogenous cohort, we focused specifically on the 18 NSCLC patients receiving LITT + ICB to 19 lesions and compared these to 25 patients who received LITT alone or in combination with targeted therapy (LITT only) to 26 NSCLC brain metastases. One NSCLC patient in the LITT + ICB group received treatment for 2 separate lesions >6 months apart, while one patient in the LITT-only group received LITT to two lesions during the same procedure. Median follow-up was 20.3 months for the LITT + ICB group and 66.6 months for the LITT-only group.

Demographic data are shown in **Table 1**. The median age in the LITT + ICB group was 61.5 (range 47–74) years; 10 (55.6%) were female and 13 (72.2%) were non-Hispanic White. The median KPS for patients receiving a combination of LITT + ICB was 90 (70–100). Age, gender, race, baseline KPS, and primary histology (adenocarcinoma, squamous cell carcinoma, or other) did not differ between groups; 100% of patients in the LITT + ICB-group and 96% of patients in the LITT-only group had KPS ≥70.

**Table 1.** Baseline Clinical Data

Patient characteristic	LITT + ICB (*n* = 18)	LITT only	*P*-value
		(*n* = 25)	
Median age (range)	61.5 (47–74)	66 (38–80)	.28
Gender, *n* (%)			.58
Male	8 (44.4)	9 (36.0)	
Female	10 (55.6)	16 (64.0)	
Non-Hispanic white, n (%)	13 (72.2)	15 (60.0)	.41
Median KPS (range)	90 (70–100)	90 (60–100)	.82
Histology, n (%)			.22
NSCLC—adenocarcinoma	13 (72.2)	23 (92.0)	
NSCLC—squamous	2 (11.1)	1 (4.0)	
NSCLC—other	3 (16.6)	1 (4.0)	
Systemic burden of disease at time of LITT, *n* (%)			.29
Absent, NED	0 (0)	4 (16.0)	
Stable disease	12 (66.7)	12 (48.0)	
Progressive disease	5 (27.8)	8 (32.0)	
Unknown	1 (5.6)	1 (4.0)	
Multiple lesions at the time of LITT? *n* (%)			.48
Yes	15 (83.3)	18 (72.0)	
No	3 (16.7)	7 (28.0)	
PD-L1 testing? *n* (%)			**.0426**
TPS ≥ 50%	2 (11.1)	2 (8.0)	
TPS 1–49%	7 (38.9)	2 (8.0)	
TPS < 1%	2 (11.1)	2 (8.0)	
Unavailable	7 (38.9)	19 (76.0)	
TMB testing? *n* (%)			**.0264**
TMB ≥ 10/Mb	6 (33.3)	2 (8.0)	
TMB < 10/Mb	1 (5.6)	0 (0)	
Unavailable	11 (61.1)	23 (92.0)	
Driver mutation? *n* (%)			**.0021**
EGFR	1 (5.6)	4 (16.0)	
ALK	0 (0)	4 (16.0)	
KRAS	7 (38.9)	0 (0)	
No driver	9 (50.0)	13 (52.0)	
Unavailable	1 (5.6)	4 (16.0)	
Targeted therapy (*n)*	-	Osimertinib (4), Afatinib (3), Alectinib (2),	—
		Lorlatinib (2), Ceritinib (1)	
Immunotherapy (*n*)	Pembrolizumab (8), Nivolumab (5), Atezolizumab (2), Durvalumab (2),	—	—
	Ipilimumab/Nivolumab (1)		
Lesion characteristic	LITT + ICB,	LITT only,	*P*-value
	NSCLC	NSCLC	
	(*n* = 19)	(*n* = 26)	
Median preoperative diameter (cm, range)	2.2 (1.5–3.7)	2.3 (1.4–3.7)	.22
Lesion location, *n* (%)			.64
Frontal	10 (52.6)	16 (61.5)	
Parietal	4 (21.1)	3 (11.2)	
Temporal	2 (10.5)	5 (19.2)	
Occipital	2 (10.5)	2 (7.7)	
Deep	1 (5.3)	0 (0)	
Cerebellar	0 (0)	0 (0)	
Previous SRS to index lesion, *n* (%)	19 (100.0)	26 (100.0)	—
Previous WBRT to index lesion, *n* (%)	3 (15.8)	2 (8.0)	—
Previous surgery to index lesion, *n* (%)	3 (15.8)	5 (19.2)	—
Median time from index SRS to LITT, months (range)	12.7 (4.4 -43.0)	10.6 (5.2 – 81.3)	.8

Bolded *P*-values are significant.

Abbreviations: NED, no evidence of disease; SRS, stereotactic radiosurgery; WBRT, whole brain radiation therapy, TPS, tumor proportion score; TMB, tumor mutational burden.

Most patients had stable or absent extracranial disease at the time of LITT (66.7% of LITT + ICB vs. 64.0% of LITT-only patients), and almost all had multiple intracranial lesions at the time of LITT (83.3% of LITT + ICB and 72.0% of LITT-only). Positive PD-L1 staining (*P* = .0426) and tumor mutational burden elevation (*P* = .0264), as well as KRAS mutations, were more frequent in the LITT + ICB group, while tumors were more frequently driven by EGFR or ALK in the LITT-only group (*P* = .0021). No tumors had ROS1 mutations. Consequently, 12 of the 25 (48.0%) LITT-only patients were on oral small-molecule inhibitors, of which osimertinib was most common (4 patients); the remainder were not on systemic therapy within 6 weeks of LITT. In the LITT + ICB cohort, pembrolizumab was most frequently used (8 patients), followed by nivolumab (5), atezolizumab (2), durvalumab (2), and combination ipilimumab/nivolumab in 1 patient. Fifteen of 18 NSCLC patients in the LITT + ICB group were already receiving ICB before undergoing LITT, while ICB was initiated post-LITT in just 3 cases. For patients on ICB pre-LITT with available data, the median number of cycles at the time of LITT was 11 (5–45, *n* = 13).

**Figure 1.**
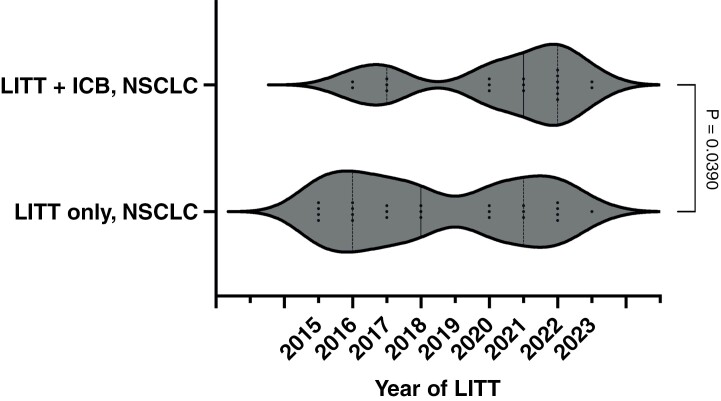
Year of laser interstitial thermal therapy (LITT) treatment. Violin plots of patients receiving LITT ± immune checkpoint blockade (ICB) in each group. Non-small cell lung carcinoma patients receiving or not receiving ICB were compared using the Mann–Whitney U test, *P* = .0390.

Lesion-specific characteristics are displayed in **Table 1**. The median preoperative lesion diameter did not differ between groups (2.2 vs. 2.3 cm for LITT + ICB vs LITT only, respectively, *P* = .2182). Across groups, the majority of lesions were located in the frontal lobe, and all had previously been treated with SRS. The median time between the SRS and LITT to a target lesion was 12.7 (4.4–43.0) months in the LITT + ICB group and 10.6 (5.2–81.3) months in the LITT-only group, *P* = .80. Five lesions total (3 LITT + ICB and 2 LITT-only; 3/5 cases at an outside institution) had received prior WBRT, with 3 LITT + ICB and 5 LITT-only patients having also undergone resection of the lesion of interest. Notably, NSCLC patients on ICB generally presented for LITT procedures in later years relative to the LITT-only group (**Figure 1**, median 2021 vs. 2018, *P* = .039).

### Procedural Outcomes

We next investigated the impact of ICB on typical procedural outcome measures, such as LOS, rates of non-routine discharge and complications, and 30-day readmissions (**Table 2**). The median time between the LITT procedure and the closest ICB dose in the combination treatment group was 2.29 (0.85–5.98) weeks. Interestingly, a significantly lower proportion of patients in the LITT + ICB group were on steroids in the month prior to LITT (47.4% vs. 82% of patients, *P* = .0098). Also of note, there were slightly lower rates of RN found on biopsy between NSCLC patients in the LITT + ICB (11 patients, 57.9%) vs LITT only (20, 80.0%) groups (*P* = .11). Notably, only one of the two operated lesions was biopsied in the patient receiving LITT-only to 2 separate metastases. There was no difference in the median % ablation (98% for LITT + ICB vs. 95% for LITT alone) between groups (*P* = .10). Similarly, the median length of stay (LOS) (1 day for both cohorts), rate of discharge to home (100% vs 92%), intraoperative complications (0% vs. 4%), and 30-day readmissions (15.8% vs. 16.0%) did not differ for LITT + ICB vs. LITT only cases, respectively. There was no difference in the rate of post-LITT radiotherapy for patients with biopsy-proven recurrent tumors.

**Table 2.** Procedural Outcomes Following LITT

Procedure characteristic	LITT + ICB (*n* = 19)	LITT only (*n* = 25)	*P*-value
Steroids within 1 month prior to LITT, n (%)	9 (47.4)	21 (84.0)	**.0098**
Median time between ICB & LITT (range)	2.29 (0.85–5.98)	—	—
Biopsy result, *n* (%)			.11
Recurrent tumor	8 (42.1)	5 (20.0)	
Radiation necrosis	11 (57.9)	20 (80.0)	
No biopsy	0 (0)	1^a^	
Median % ablation (range)	98 (95–100)	95 (70 – 100)^a^	.10
Median length of stay (days)	1 (0–10)	1 (0 – 6)	.91
Discharge to home, *n* (%)	19 (100.0)	23 (92.0)	.50
Intraoperative complications, *n* (%)	0 (0)	1 (4.0)	—
30-day readmissions, *n* (%)	3 (15.8)	4 (16.0)	.99
Post-LITT RT for recurrent tumor, *n* (%)	6/8 (75.0)	2/5 (40.0)	.29

Bolded *P*-values are significant.

^a^In the patient who received LITT to 2 lesions during the same procedure, only one was biopsied. The unbiopsied lesion was excluded from analyses of biopsy result; both lesions were included in calculating % ablation.

Abbreviations: RT, radiotherapy.

### Post-LITT Steroid Usage

**Figure 2.**
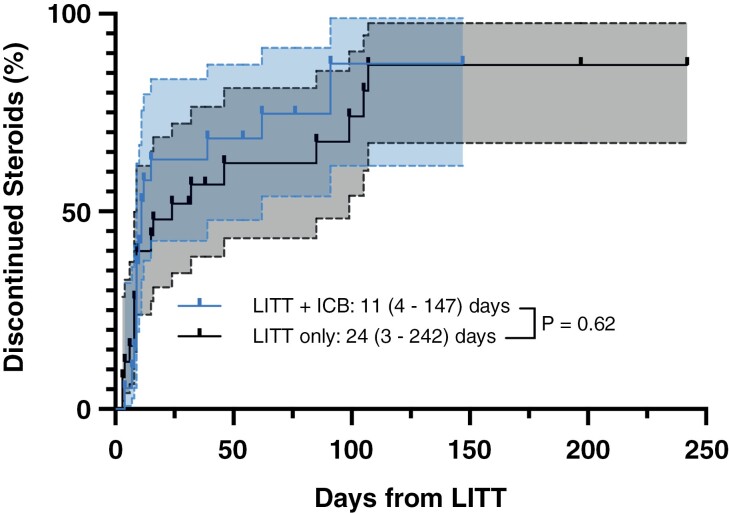
Patients receiving laser interstitial thermal therapy (LITT) with and without checkpoint blockade discontinue steroids at equal rates. Cumulative incidence curve of the probability of steroid discontinuation for ≥4 weeks following LITT with and without combination immunotherapy compared via the log-rank test. Shaded regions represent 95% confidence intervals of the plotted data. Following 15/19 (78.9%) procedures in the LITT + immune checkpoint blockade (ICB) group and 19/25 (76.0%) in the LITT alone group, steroids were successfully discontinued.

LITT is known to cause a transient postoperative increase in both total lesion volume and perilesional edema, in part due to the breakdown of the BBB and infiltration of immune cells.^3^ To manage this swelling, patients at our institution typically receive a brief, 1–2 week steroid taper post-LITT if they do not have significant new or worsened postoperative deficits. To determine whether ICB use was associated with an increased potential for symptomatic post-LITT edema, we assessed the time to successful steroid discontinuation in each group (**Figure 2**). Patients in the LITT + ICB group discontinued steroids at a median of 11 (4–147) days postoperatively, while patients in the LITT alone group discontinued them at 24 (3–242) days, *P* = .62. Steroids were successfully discontinued following 15/19 and 19/25 procedures in each group, respectively.

### Post-LITT Local Control and Survival

**Figure 3.**
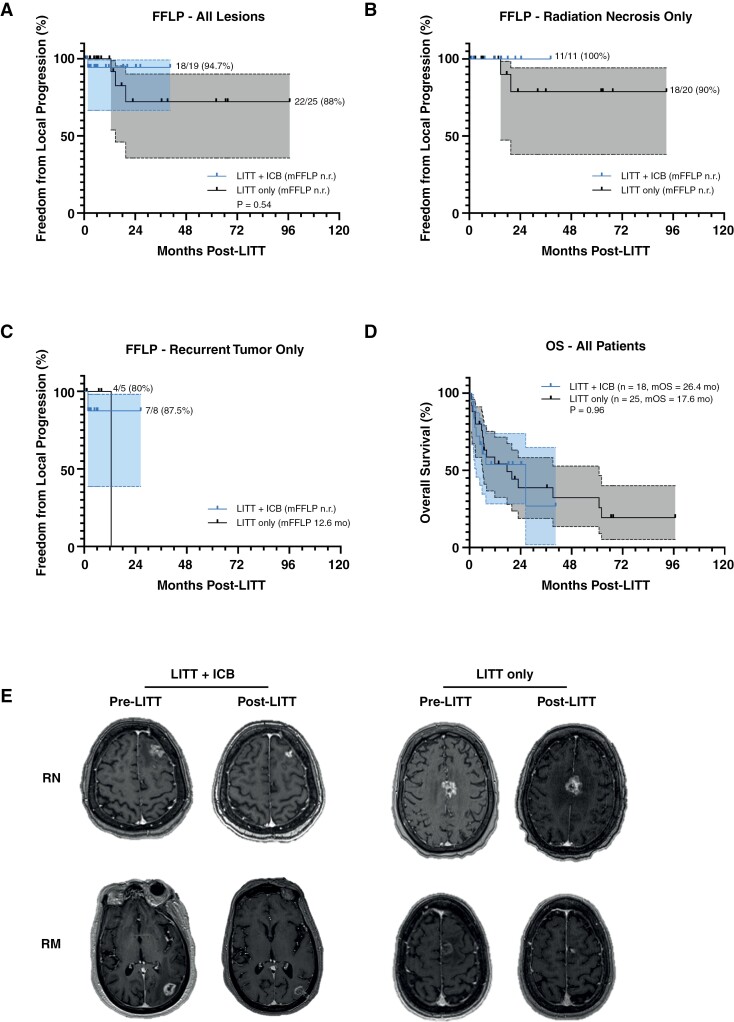
Post-laser interstitial thermal therapy (LITT) freedom from local progression and overall survival with and without immune checkpoint blockade. Kaplan–Meier curves of freedom from local progression (FFLP) in patients receiving LITT + immune checkpoint blockade (ICB) vs LITT only to (**A)** all lesions, (**B)** biopsy-proven radiation necrosis only, or (**C)** recurrent tumor only. FFLP rates at the study cutoff are shown adjacent to each curve. (**D)** Overall survival from the time of index LITT treatment to all patients in each cohort. Shaded areas represent 95% confidence intervals of the plotted data. Comparisons were made using the log-rank test where applicable. (**E)** Representative pre- and post-LITT contrast-enhanced T1-weighted images for patients in each treatment group with radiation necrosis (RN) or recurrent metastasis (RM) at the time of last follow-up or local progression.

Freedom from local progression and overall survival (OS) were assessed to determine the degree of intracranial control achieved in the study cohorts. One lesion in the LITT + ICB group locally progressed following LITT for a 94.7% FFLP rate at the time of study cutoff (**Figure 3A**). Three of the twenty-five lesions locally progressed in the LITT-only group, resulting in an 88% FFLP rate, HR 0.49, (95% CI: 0.05–4.74), *P* = .54. When evaluating only lesions with RN identified on biopsy, the local control rates were 100% versus 90% for LITT + ICB versus LITT only, respectively, (**Figure 3B**). Accordingly, the median duration of FFLP was not reached for all tumors or for biopsy-proven RN specifically. For recurrent tumors, local control at the study cutoff was 87.5% (7/8) for LITT + ICB, mFFLP not reached, and 80% (4/5) for LITT only, mFFLP 12.6 months (**Figure 3C**). Median OS was 26.4 (95% CI: 3.1–not reached) months for patients receiving LITT + ICB and 17.6 (95% CI: 6.1–60.9) months for those treated with LITT alone, HR 1.02 (95% CI: 0.44–2.35), (*P* = .96, **Figure 3D**). Representative pre- and post-LITT images at the last follow-up (local control) or local progression for patients in each group are shown in **Figure 3E**; the patient in the LITT-only group in whom pre-LITT biopsy originally showed RN ultimately progressed at 14.8 months from surgery.

### Adverse Events

To analyze whether there were any important safety signals related to the combination of ICB and LITT, we assessed patients for procedural AEs and immune-related AEs (iRAEs) during the study period (**Table 3**). There were a total of 26 AEs of any grade in the LITT + ICB NSCLC cohort and 22 in the LITT-only cohort, respectively. There were 3 Grade 3 or higher events in the LITT + ICB group and 5 in the LITT + ICB group. The most frequent AEs across groups were post-operative headache (8 LITT + ICB vs. 5 LITT-only patients) and seizure (6 LITT + ICB and 5 LITT-only patients), with one patient in the LITT-only group suffering status epilepticus following the progression of an unablated motor-cortex associated metastasis leading to death. IRAEs followed similar patterns to known AEs from prospective clinical trials, with hypothyroidism (4) and infusion-related reactions (4) noted most frequently, followed by one case each of elevated transaminases and a mild exacerbation of psoriasis in a previously diagnosed patient. There were no serious iRAEs in the study.

**Table 3.** Post-LITT Adverse Events

Adverse event	LITT + ICB, *n* = 18		LITT only, *n* = 25	
	Any grade	Grade ≥ 3	Any grade	Grade ≥ 3
Aphasia	1 (5.6)	1 (5.6)	—	—
Cognitive disturbance	—	—	2 (8.0)	1 (4.0)
Dizziness	1 (5.6)	—	—	—
Edema cerebral	1 (5.6)	1 (5.6)	—	—
Facial nerve disorder	—	—	1 (4.0)	—
Fatigue	—	—	3 (12.0)	1 (4.0)
Gait disturbance	—	—	1 (4.0)	—
Headache	8 (44.4)	—	5 (20.0)	—
Intracranial Hemorrhage	1 (5.6)	—	—	—
Memory impairment	—	—	1 (4.0)	—
Muscle weakness	4 (22.2)	—	3 (12.0)	2 (8.0)
Nausea	—	—	1 (4.0)	—
Seizure	6 (33.3)	—	5 (20)	1 (4.0)
Soft tissue infection	1 (5.6)	1 (5.6)	—	—
Visual disturbance	3 (16.7)	—	—	—
*Total events*	26	3	22	5

## Discussion

We performed the largest study to date assessing outcomes and AEs of a combination of ICB and LITT, as well as the first such study to focus on brain metastases. Our study exclusively featured patients with NSCLC, a histology FDA-approved for treatment with ICB.^24,25^ Despite the theoretical risk of increased edema with combination therapy, the procedural benefits typically associated with LITT in other clinical contexts are preserved in our cohort; patients receiving combination LITT and ICB were similar to patients receiving LITT alone with regards to shortened LOS, frequency of discharge to home, and had low rates of complications and 30-day readmissions.^1,2^ In general, iRAEs were commensurate with those seen in prospective studies, with no serious iRAEs and infrequent CNS AEs.^15,25,26^ While our study was underpowered to detect clinically meaningful survival differences, there did not appear to be a survival decrement in the context of LITT + ICB. Furthermore, despite a higher percentage of biopsy-confirmed brain metastases in the combination therapy (42.1%) versus the LITT-only group (20.0%), both treatment arms experienced excellent post-LITT local control. These analyses provide encouraging initial safety data on this treatment combination and may hint at the ability of checkpoint inhibition and in situ vaccination with LITT to augment antitumor T cell responses, serving as a baseline for future studies.

Perhaps most interestingly, while most patients did not have a pre-LITT steroid requirement in the LITT + ICB cohort, both treatment arms were able to rapidly discontinue steroids following LITT. It is possible that paired administration of these 2 intracranially active and pro-inflammatory treatment modalities might have augmented the post-treatment edema that is characteristic of LITT and may even serve as a biomarker of potential efficacy; accordingly, future radiomic or volumetric analyses of LITT-treated lesions may ultimately better quantify this effect.^27–30^ Notably, while concern has existed that the use of dexamethasone and other steroids may blunt the effects of ICB, their use in the perioperative period is commonplace in neurosurgery and likely necessary following LITT.^19,20^ Indeed, awareness of the negative effect of steroids on the anticancer efficacy of ICB may have partially driven the apparent avoidance of preoperative steroids in the ICB arm of the study relative to the LITT alone arm. Future studies examining the peripheral immune response to combination treatment as well as to LITT alone are needed to better understand how best to use steroids in this context, as well as whether there is a role for steroid-sparing agents, such as bevacizumab or small molecule inhibitors of the receptor for advanced glycation end products (RAGE).^15,31^

In parallel with the development and subsequent adoption of checkpoint inhibitors, the concept of the CNS as “immunologically privileged” has shifted to an understanding of the brain and other CNS constituent tissues moreso as immunologically distinct niches.^10,32–35^ In this context, the efficacy of ICB against brain metastases for specific solid tumor types is valuable evidence regarding potential antitumor immune responses in the brain.^19,36–39^ Nonetheless, both primary brain and brain metastatic tumors employ unique mechanisms to engender local and peripheral immunosuppression in manners not seen amidst extracranial solid tumors.^9,10^ LITT-treated, non-resected necrotic tumors may permit persistence and presentation of residual neoantigens that facilitate in situ vaccination and epitope spreading that can be further potentiated by checkpoint blockade; furthermore, local hyperthermia has been associated with clearing of and resistance to the development of extracranial metastases through apparent T-cell mediated abscopal effects.^3,7^ For glioma, two early phase trials have thus far compared ICB + LITT to ICB alone, despite phase 3 evidence of a lack of efficacy of ICB monotherapy for primary tumors.^11–13,15,16^ Furthermore, the absence of a cytoreductive control arm (eg, LITT alone or resection), which is strongly detrimental to survival, renders definitive conclusions challenging.^40^ A trial comparing neoadjuvant versus adjuvant ICB in combination with LITT is currently ongoing (NCT03277638). In contrast, the present study controls for the cytoreductive effect of LITT and provides foundational evidence for whether promoting an antitumor immune response with ICB in a responsive tumor type (NSCLC) is safe and feasible.

### Limitations

The primary purpose of this retrospective, single-center study was to demonstrate our experience with a combination of ICB and LITT, with a focus on safety and procedural outcomes in CNS metastases. While we elected not to conduct an artificially matched comparison between the NSCLC groups, our analyses showed they were comparable at baseline, with similar clinical characteristics (age, KPS, time from upfront therapy), systemic and intracranial disease status, and underlying lung cancer histology. Notably, the more recent adoption of ICB in oncology is reflected in the later treatment years of patients receiving LITT + ICB versus those receiving LITT alone. Despite providing encouraging safety data, our study did not detect a difference in OS, which likely reflects sample size, slightly limited follow-up in the LITT + ICB group, and current treatment paradigms for NSCLC. For instance, this study was conducted in patients with NSCLC, in which targeted therapies are typically given upfront should a patient have a molecular alteration in *EGFR*, *ALK,* or *ROS1.* Accordingly, patients in the LITT + ICB group may have presented without targetable molecular alterations or have been receiving ICB as part of a later line of therapy.^41^ Finally, the optimal duration of ICB remains an open question in oncology, and it is possible that synergy between LITT and ICB may be highest when immunotherapy is first initiated—though upfront LITT is not currently indicated for CNS metastases—which should be assessed in a prospective trial.^42,43^

## Conclusion

We performed the first study examining a combination of LITT + ICB in patients with brain metastases, and the largest such study to date of patients with CNS tumors more generally. The combination of ICB and LITT did not lead to increased LOS or increased frequency of non-routine discharge, complications, or readmissions. Likewise, it was not associated with a decrement in survival. Adverse event frequency was similar between patients receiving LITT + ICB versus LITT alone. Despite differences in baseline steroid use, patients receiving combination therapy successfully discontinued steroids following LITT. These data provide an important baseline of safety and feasibility for promising future investigations combining laser ablation and immunotherapy.

## Data Availability

Deidentified clinical data is available upon reasonable request in accordance with institutional data-sharing policies.
